# Aircraft-Assisted Pilot Suicides in the General Aviation Increased for One-Year Period after 11 September 2001 Attack in the United States

**DOI:** 10.3390/ijerph15112525

**Published:** 2018-11-12

**Authors:** Alpo Vuorio, Tanja Laukkala, Ilkka Junttila, Robert Bor, Bruce Budowle, Eero Pukkala, Pooshan Navathe, Antti Sajantila

**Affiliations:** 1 Mehiläinen Airport Health Centre, 01530 Vantaa, Finland; 2 Department of Forensic Medicine, University of Helsinki, 00014 Helsinki, Finland; antti.sajantila@helsinki.fi; 3 Mehiläinen Kielotie Health Centre, 01300 Vantaa, Finland; tanja.laukkala@duodecim.fi; 4 Faculty of Medicine and Life Sciences, University of Tampere, Finland and Fimlab Laboratories, 33014 Tampere, Finland; ilkka.junttila@uta.fi; 5 Royal Free Hospital, Pond Street, London NW3 2QG, UK; robertbor@hotmail.com; 6 Centre for Aviation Psychology, London NW3 1ND, UK; 7 Center for Human Identification, University of North Texas Health Science Center, 3500 Camp, Bowie Blvd, Fort Worth, TX 76107, USA; bruce.budowle@unthsc.edu; 8 Faculty of Social Sciences, University of Tampere, 33014 Tampere, Finland; Eero.Pukkala@cancer.fi; 9 The Maitland Hospital, Maitland 2320, Australia; Pooshan.Navathe@hnehealth.nsw.gov.au

**Keywords:** September 11 terrorist attacks, pilot aircraft-assisted suicide, copycat effect

## Abstract

Pilot aircraft-assisted suicides (AAS) are rare, and there is limited understanding of copycat phenomenon among aviators. The aim of this study was to evaluate the possible effect the 11 September 2001, terrorist attacks had on pilot AASs in the U.S. Fatal aviation accidents in the National Transportation Safety Board (NTSB) database were searched using the following search words: “suicide”, “murder-suicide” and “homicide-suicide”. The timeline between 11 September 1996, and 11 September 2004, was analyzed. Only those accidents in which NTSB judged that the cause of the accident was suicide were included in the final analysis. The relative risk (RR) of the pilot AASs in all fatal accidents in the U.S. was calculated in order to compare the one, two, and three-year periods after the September 11 terrorist attacks with five years preceding the event. The RR of a fatal general aviation aircraft accident being due to pilot suicide was 3.68-fold (95% confidence interval 1.04–12.98) during the first year after 11 September 2001, but there was not a statistically significant increase in the later years. This study showed an association, albeit not determinate causal effect, of a very specific series of simultaneous terrorist murder-suicides with subsequent pilot AASs.

## 1. Background

Copycat phenomenon (suicidal behavior provoked by media exposure) in pilot aircraft-assisted suicides (AASs) has been studied since the 1970s with inconclusive results [1,2], while longitudinal case series on pilot AASs have shown the difficulty of predicting rare events [3,4].We have previously analyzed pilot AASs after the Germanwings pilot murder-suicide incident [5], with no increase in pilot AASs was observed in the U.S. or Germany during a two-year period after the incident as compared to the previous five-year period.

The negative impact of exposure to atrocities on mental health is well documented, from trauma and-stress related disorders, to the level of copycat suicides [6,7,8,9]. The exposure to traumatic events through media alone is sufficient to cause psychological distress or even stress-related disorders [10]. Copycat behavior can be defined as suicidal behavior provoked by media exposure, also described as the “Werther effect” (from Goethe’s novel Die Leiden des Jungen Werthers). In addition, the term “Papageno effect” has been proposed to describe protective media measures [7,11]. The name originally refers to a bird catcher character from Mozart’s Magic Flute, who became suicidal but recovered after his friends intervened [7,11]. World Health Organization (WHO) has guidance on responsible media reporting after suicide, and recent National Institute for Health and Care Excellence (NICE) guidelines on suicide prevention have also focused on the impact of media [12,13].

Pilots are generally regarded as being psychologically resilient and healthier than the general population due to initial health assessment of specialized aeromedical examiners, which excludes individuals with severe risk factors for suicidal behavior, such as repeated suicide attempts or current major depression. Mandatory health assessments are regularly repeated, as requested in aviation authorities’ guidance on aviation medical examiners (AME)s [14,15]. At an individual pilot level, mental health fitness assessments may have an impact on aviation safety [16]. However, the use of several aircrafts in the September 11 attacks focused attention internationally on the actions of aviators and also on the potential vulnerability of aviation safety with excessive and repeated media coverage. In relation to military pilots, the U.S. Air Force has published fairly constant suicide rates for that time period in an analysis of the U.S. Air Force suicide prevention program [17].

A study by Claassen et al. [18] has shown no increase in suicide rates in the general population in areas surrounding the three airline crash sites in New York, the Pentagon in Washington D.C, and Somerset County in Pennsylvania after 11 September 2001. Claassen et al. [18] concluded that geographical proximity is less important compared to other event characteristics and more research is needed regarding relevant social factors. This is in line with the study of Mezuk and coworkers [19], who reported no increase in New York suicide rates after the attack. Monthly homicide statistics in the New York City area did not reveal an increase in homicide or suicide rates after 11 September 2001 [20,21]. 

Although Pridemore and colleagues [21] showed that the rate of homicide or suicide deaths in New York was not increased following the attacks of 11 September 2001, Jordan et al. [22] showed that the standardized mortality ratio (SMR) for suicide was elevated (SMR = 1.82) among rescue recovery workers, while SMR for all-cause mortality was not elevated (SMR = 0.69). In another study of mortality among World Trade Center rescue workers, Stein et al. [23] reported a lowered all-cause and cause-specific mortality among rescue workers during a time period of 2002–2011 and did not find any association relating to the duration of rescue work and mortality. These long-term follow-up study results are not conclusive, inviting more analyses among different professional groups related to similar types of incidents. Starkman [24] has reported a one-year increase in suicide attempts after 11 September 2001 in Michigan, U.S, and the effect was greatest in the months following the attacks.

One aspect to explore is the research on suicidal ideation. In a survey of 871 adults who experienced loss during the September 11 attacks, individuals with complicated grief had significantly high rates of suicidal ideation even after adjusting to comorbid depression [25]. Additionally, suicidal ideation was increased in another primary care cohort after 11 September 2001 (*N* = 444) in Manhattan [6].

The aim of this study is to evaluate possible changes in pilot aircraft-assisted suicides after the 11 September 2001, terrorist murder–suicides in the U.S.

## 2. Methods

The U.S. National Transportation Safety Board (NTSB) database was searched on 26 June 2018, using the following search words: “suicide”, “murder–suicide”, and “homicide–suicide” [26]. Fatal aviation accidents in the U.S, with full formal accident investigation reports finalized at the time of the search and the cause of accidents assessed as pilot suicide in the NTSB accident investigation, were included as index cases to this study. These NTSB accident investigations were further analyzed in-depth to assess the descriptive factors in these suicide processes. Only those accidents in which NTSB judged that the cause of the accident was suicide were included in the statistical analysis.

Frequency of the aircraft-assisted pilot suicides out of all fatal accidents in the U.S was calculated for the reference period (5 years before the 11 September 2001 terrorist murder-suicides event) and separately for the first, second, and third year after the September 11 terrorist attacks. The relative risk (RR) of the likelihood of an aircraft accident being due to pilot suicide for the three post-attack years was calculated.

## 3. Results

The number of fatal aviation accidents and relative risks of the likelihood of pilot AAS being due to suicide three years after 11 September 2001, calculated for each year separately, is presented in Table 1. All pilots who died were male. The RR was 3.68 in the first year and decreased close to 1.0 in the third year. Only the observation for the first year was statistically significant.

**Figure 1:**
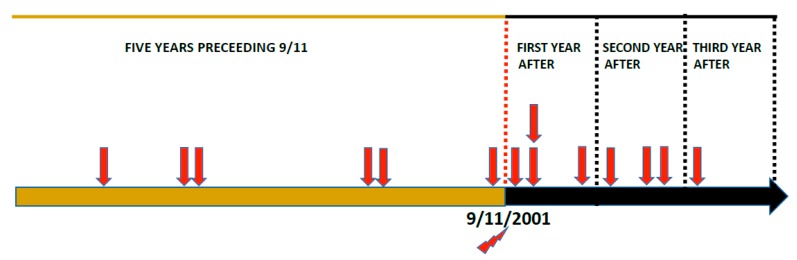
Timeline of aircraft-assisted pilot suicides five years before and three years after 11 September 2001. Red arrows indicate aircraft assisted suicides.

Altogether 23 fatal aviation accident reports were obtained with the specified search words. All index cases were identified with the search word “suicide”, search words “homicide-suicide”, or “murder-suicide” did not yield any additional index case incidents. Fourteen of these incidents were caused by a pilot’s or co-pilot’s suicidal act according to NTSB accident investigations, and these incidents are described in detail in Table 2. All the incidents were operationally related to general aviation, although three pilots had also Class I (commercial pilot) medical certification even though they were not flying commercial aircraft at the time of the suicide crash. It should be noted that three of the deceased pilots were also flight instructors. Altogether fourteen pilots or student pilots died in these incidents. Their ages ranged from 15 to 69 years. Eight of these aircraft-assisted suicides occurred after 11 September 2001; four within the first year, three during the second year, and one in the third year, while six aircraft-assisted suicides took place during the five-year-period before it (see timeline in Figure 1). NTSB causes of death for excluded cases in this search were: four undetermined causes of fatal aviation accidents, two related to psychological problems (drugs or alcohol were mentioned at least as a contributing factor), in two reports weather conditions were mentioned, and in one case death occurred due to a passenger’s suicidal act.

## 4. Discussion

The one-year RR of 3.68 of aircraft-assisted pilot suicides in the U.S. after 11 September 2001, is in line with the results of rescue worker’s suicide mortality reported by Jordan et al. [22], and some earlier copycat reports by Fink et al. [8]; see also Sisask and Värnik [7]. While fatal aviation accidents are rare, large or long-term register studies such as the study of Blettner et al. [27] and Politano and Walton [3] enable assessments to detect plausible effects of such events. General population suicide rates have been relatively stable during the time period of this study, but have increased in the U.S. during the last few years [28,29]. 

In studies from other countries, in England and Wales no increase of suicides was observed after 11 September 2001 [30] and there was no detectable change in the suicide rates in the Germany study [31]. An analysis of suicide rates from the Netherlands by de Lange and Neeleman [32] showed evidence of an increase in the suicide rates in the weeks after 11 September. Regarding trauma and stress related disorders, a Danish study by Hansen et al. [33] reported an increase in trauma and stress -related disorders after 11 September 2001, in line with several U.S. studies [34,35].

In one suicide flight involving a young pilot after 11 September 2001, the plane crashed intentionally into a high office building. According to the report there was a suicide note but it was not included in the accident investigation data. The helicopter pilots who intercepted this airplane attempted to signal the young pilot to land. According to the helicopter pilots, the student pilot saw their hand gestures and gestured back, but they could not determine the meaning of the gestures. Investigation of this incident led to a Federal Aviation Administration security notice release.

In the incidents investigated in this study, previous depression with suicide attempts was described in one case, ongoing treatment for depression in two cases, and a history of substance abuse and arson conviction in one case. Toxicology revealed that in six of these incidents there were medications or substance misuse not compatible with flying according to FAA. Some pilots with no information on previous psychological or psychiatric issues had recent stressful personal situations, such as legal or interpersonal difficulties.

The choice of crashing an aircraft to commit suicide among some pilots may in part be understood through the desire to combine passion for flying with death, reflecting an intimate bond between the pilot and his means of dying. Occasionally there may also be a hope that the incident would be construed as a medical fatality if the evidence were to be destroyed in the crash (for instance if the aircraft disappears).

One of the Class 1 pilots had a history of substance dependence. He was convicted of driving when intoxicated on several occasions and of arson, and had acute stressors. He left a suicide note. Another Class 1 pilot with no known immediate stressful events or health-related issues asked air traffic control to call for airport rescue and firefighting, and “also if you could tell my family and friends that I love them very much”. Thus, he could be reached immediately before the lethal act. The third Class 1 pilot with military helicopter experience told the dispatcher he was going to meet someone. At no time did he display any signs of stress or unusual behavior. There were no records of previous incidents, and he had flight experience with various helicopters. This description gives an impression of a person who made a decision and enacted it; he was not in contact with any air traffic control facility at the time of the incident.

Previous suicide attempts and major depression are risk factors for completed suicide, but data on depression among aviators is limited [13,36]. In civil aviation, ongoing symptomatic major depression is not compatible with flying duties. After full remission and sufficient follow-up, International Civil Aviation Organization (ICAO) and aviation authorities currently accept psychological and some pharmacological treatments for aviators to prevent recurrence [14,15].

In aviation, full remission of depressive or stress-related symptoms, good compliance, and a reasonable follow-up time post-recovery are a prerequisite to the consideration of an aviator’s return to flying duties [13,14,15,36,37,38]. Accepted antidepressants used to prevent symptoms are assessed through specific programs that focus on comprehensively assessing fitness to fly, symptoms, treatment adherence, and especially aero-medically relevant side-effects (e.g., fatigue).

In relation to mental health and suicide, it should be noted that among pilots regular health examinations contribute to the recognition of risk factors of suicidal behavior. The actual risk factors for pilots may somewhat differ from the general population, in relation to the role of any defined psychiatric disorders [13,39,40].

The limitations of this study include the fact that our data are based on information in the accident investigations, and we do not have access to additional medical information for determining aviator suicides nor to any information on aviators’ attempted suicides prior to the incidence. The data are reliant on NTSB reports, and these reports are incomplete in some cases and certain details remain unknown. The news information after 11 September and timing of news delivery was not analyzed, but there are several studies from the U.S. on psychological effects after 11 September 2001 [6,25,41]. We do not think that any flying pilot in the U.S. could have avoided the news, due to immediate alerts and airspace closures in the U.S. The role of media research and balancing Werther and Papageno effects in general population suicide prevention is more thoroughly assessed elsewhere [42,43,44,45]. However, we consider our pilot AAS estimates to be conservative since those fatal aviation incidents where autopsy indicated suicide, but NTSB accident investigations did not agree, were excluded. 

## 5. Conclusions

This study showed an association of 11 September 2001, with pilot AASs. The copycat effect was present for one year after 11 September 2001. The causal factors behind this statistical association remain unclear in context to the theoretical approaches on suicidality. The use of aircraft as the means to commit the terrorist attacks in the U.S. on 11 September 2001, may have had a negative effect on a few acutely vulnerable pilots. This vulnerability warrants further investigation, particularly with reference to the copycat phenomenon and it needs to be taken into account in aviation medical safety risk assessments.

## Figures and Tables

**Table 1:** The number and the risk ratios of pilot aircraft-assisted suicides five years before and three years after 11 September 2001, in the U.S.

Follow-Up Period	Dates	No. of Suicides	No. of Fatal Aviation Accidents	FRQ	Risk Ratio	95% Confidence Interval
Reference	11 September 1996–10 September 2001	6	1861	0.32%	1.00	Reference
1st year	12 September 2001–11 September 2002	4	337	1.19%	3.68	1.04–12.98
2nd year	12 September 2002–11 September 2003	3	375	0.80%	2.48	0.62–9.88
3rd year	12 September 2003–11 September 2004	1	354	0.28%	0.88	0.11–7.26

FRQ = frequency.

**Table 2:** Aircraft-assisted pilot suicides in the National Transportation Safety Board database five years before and three years after the 11 September 2001.

Event Date and State	Medical Certificate * (Assessment)	PM Toxicology	Health before the Flight	Cause of Accident by NTSB **	Other Information on Events before the Incident Flight
16 September 2003, Georgia	Class 2 (14 May 2002)	Ethanol	Normal	Intentional suicidal flight into the ground while impaired by alcohol.	Pilot threatened to commit suicide by flying into a mountain. No suicide note.
21 July 2003, Minnesota	Class 3 (9 April 2003)	Citalopram, fluoxetine, diphenhydramine	Depression	An act of suicide.	Suicide attempt with medication within a week with psychiatric hospitalization, “he wanted to get the courage to kill himself”, left the hospital a day before the accident flight. No suicide note.
25 February 2003, Florida	Class 1 (12 March 2002)	Toxicology negative	No data	The intentional suicidal act.	No data. Autopsy: Manner of death suicide. No suicide note.
17 November 2002, Texas	Class 2 expired (3 September 1987)	Toxicology not done	Depression	An intentional suicide by exiting from the airplane, contributory was psychological condition.	Friends and co-workers: ongoing treatment for depression and a recent intent to take his own life by using an aircraft. A suicide note.
12 August 2002, Nebraska	Class 2 (7 July 1998)	Toxicology done, negative	No data	The pilot’s intentional flight into terrain in an act of suicide.	On-going criminal investigation and was told to have threatened to kill himself by intentionally crashing an airplane before he would go to jail. No suicide note.
5 January 2002, Florida	Class 3 (17 November 2001)	Toxicology done, negative	No data	The pilot’s unauthorized use of an aircraft for the purpose of committing suicide.	The airplane impacted the office building at the 28th-floor level. A suicide note.
5 January 2002, Colorado	Class 2 (28 September 2000)	Venlafaxine and its metabolite	Depression	Intentional suicidal flight into terrain. Contributing was depressive state, and inappropriate medication.	Received psychotherapy for severe depression. Told that if he killed himself, he would crash a plane with only himself in it. No suicide note. Manner of death: suicide.
4 January 2002, California	Class 3 (30 March 2001)	Done, no data given	No data	Intentional flight into terrain in an act of suicide.	Subject of a criminal investigation, the pilot’s computer was seized from his home in the accident day. Additional emotional earlier distress. Coroner: suicide. No suicide note.
25 August 2001, New Hampshire	Class 3 (15 March 2000)	Done, no data given	No data	Suicide, the pilot intentionally crashed his airplane into his house.	A day before the pilot was issued a restraining order at his home and was escorted off his property. Cause of death: suicide. No suicide note.
2 October 2000, South Dakota	Class 1 (17 December 1999)	Toxicology negative	No data	Suicide.	Manner of death: suicide. No suicide note.
3 July 2000, Alaska	Class 1 (2 March 1999)	Ethanol, diazepam, cocaine, and their metabolites	Substance abuse history	Suicide.	A history of substance abuse, previously convicted of arson, sought by police. Friend: On the drive to the airport restless, agitated, depressed. Suicide note. Manner of death suicide.
11 October 1998, Oklahoma	Class 3 (7 August 1997)	Diazepam and its metabolites	No data	Suicide.	The accident site was located adjacent to a church where a friend, reported to have declined pilot’s marriage proposal the night before, was attending services. No suicide note.
6 September 1998, Florida	Class 2 (31 January 1998)	Measured ethanol level referred to ethanol consumption before the incident	No data	The pilot’s use of the aircraft to commit suicide.	Suicide note, “I do not want to live”. Manner of death: suicide.
24 November 1997, California	Class 3 (4 November 1996)	Ethanol, postmortem production	Coroner: previous heart attack	Act of suicide by intentionally diving the aircraft into the ocean.	Girlfriend contacted the operator asking if the pilot had taken off and expressed concern about suicidality. A recent mother’s death, worried about health. A will dated day before the incident, no suicide note. Mode of death: suicide.

* Class 1 = Airline Transport Pilot; 2 = Commercial Pilot; 3 = Private or Recreational Pilot; ** NTSB = National Transportation Safety Board.

## Abbreviations

AAS	Aircraft Assisted Suicide
AME	Aviation Medical Examiner
CAA	Civil Aviation Authority
CI	Confidence Interval
FAA	Federal Aviation Administration
ICAO	International Civil Aviation Organization
NTSB	National Transportation Safety Board
PM	Post-Mortem
RR	Relative Risk
